# Serological assays for severe acute respiratory syndrome coronavirus 2 (SARS-CoV-2), March 2020

**DOI:** 10.2807/1560-7917.ES.2020.25.16.2000421

**Published:** 2020-04-23

**Authors:** Ranawaka APM Perera, Chris KP Mok, Owen TY Tsang, Huibin Lv, Ronald LW Ko, Nicholas C Wu, Meng Yuan, Wai Shing Leung, Jacky MC Chan, Thomas SH Chik, Chris YC Choi, Kathy Leung, Kin Ho Chan, Karl CK Chan, Ka-Chi Li, Joseph T Wu, Ian A Wilson, Arnold S Monto, Leo LM Poon, Malik Peiris

**Affiliations:** 1 School of Public Health, Li Ka Shing Faculty of Medicine, The University of Hong Kong, Hong Kong SAR, China; 2 Contributed equally to the research; 3 HKU-Pasteur Research Pole, Li Ka Shing Faculty of Medicine, The University of Hong Kong, Hong Kong SAR, China; 4 Infectious Diseases Centre, Princess Margaret Hospital, Hospital Authority of Hong Kong, Hong Kong SAR, China; 5 Department of Integrative Structural and Computational Biology, The Scripps Research Institute, La Jolla, California, United States; 6 The Skaggs Institute for Chemical Biology, The Scripps Research Institute, La Jolla, California, United States; 7 Department of Epidemiology, University of Michigan School of Public Health, Ann Arbor, Michigan, United States

**Keywords:** COVD19, SARS-CoV-2, ELISA, receptor binding domain, serology, neutralization

## Abstract

**Background:**

The ongoing coronavirus disease (COVID-19) pandemic has major impacts on health systems, the economy and society. Assessing infection attack rates in the population is critical for estimating disease severity and herd immunity which is needed to calibrate public health interventions. We have previously shown that it is possible to achieve this in real time to impact public health decision making.

**Aim:**

Our objective was to develop and evaluate serological assays applicable in large-scale sero-epidemiological studies.

**Methods:**

We developed an ELISA to detect IgG and IgM antibodies to the receptor-binding domain (RBD) of the spike protein of severe acute respiratory syndrome coronavirus 2 (SARS-CoV-2). We evaluated its sensitivity and specificity in combination with confirmatory microneutralisation (MN) and 90% plaque reduction neutralisation tests (PRNT_90_) in 51 sera from 24 patients with virologically confirmed COVID-19 and in age-stratified sera from 200 healthy controls.

**Results:**

IgG and IgM RBD ELISA, MN and PRNT_90_ were reliably positive after 29 days from illness onset with no detectable cross-reactivity in age-stratified controls. We found that PRNT_90_ tests were more sensitive in detecting antibody than MN tests carried out with the conventional 100 tissue culture infectious dose challenge. Heparinised plasma appeared to reduce the infectivity of the virus challenge dose and may confound interpretation of neutralisation test.

**Conclusion:**

Using IgG ELISA based on the RBD of the spike protein to screen sera for SARS-CoV-2 antibody, followed by confirmation using PRNT_90_, is a valid approach for large-scale sero-epidemiology studies.

## Introduction

A novel coronavirus, severe acute respiratory syndrome coronavirus 2 (SARS-CoV-2), emerged in Wuhan, China, to cause an epidemic of severe pneumonia, now called coronavirus disease (COVID-19), which spread to other parts of China and to the rest of the world [1]. The disease is now a pandemic with, as at 22 April 2020, more than 2.4 million confirmed cases reported to the World Health Organization (WHO) from multiple continents, leading to more than 162,000 deaths [2]. While there are estimates of disease severity and infection attack rates, the true severity of disease remains a major knowledge gap because mild or asymptomatic infections are difficult to estimate [3]. The invisible ‘iceberg’ of mild infections needs to be estimated to fully assess disease severity, as was done during the 2009 influenza A(H1N1)pdm09 pandemic using population-based sero-epidemiology [4]. These studies allowed us to accurately estimate the true age-specific hospitalisation rates, intensive care admission rates and deaths [5]. Such information is crucial in order to assess development of herd immunity and to calibrate our response to this pandemic.

In order to carry out age-stratified population-based sero-epidemiology, it is important to validate serological methods that can be used in such large-scale studies. Ideally, we need highly sensitive high-throughput assays for rapid screening large numbers of sera and highly specific assays that can be used to confirm those sera identified to be positive in the screening tests. These assays also need to be evaluated in different specimen types (e.g. serum, plasma) to maximise the available options for study design. We developed an ELISA assay based on the recombinant receptor-binding domain (RBD) of the SARS-CoV-2 spike protein for use as a screening assay and micro-neutralisation (MN) and plaque reduction neutralisation tests (PRNT) using live virus in biosafety level 3 containment as confirmatory tests. We evaluate the sensitivity and specificity of each of these tests in a cohort of patients with virologically confirmed COVID-19 disease and in an age-stratified set of control sera collected before the emergence of COVID-19 to serve as a negative control population.

## Methods

### Recruitment of patients and specimen collection

Patients with COVID-19 disease RT-PCR-confirmed at the Infectious Disease Centre of the Princess Margaret Hospital, Hong Kong, were invited to participate in the study after providing informed consent. All patients who consented to participate were included in the study. 

The severity of the patients was categorised as follows. (i) mild: no sign of pneumonia on X-ray or computerised tomography imaging, mild clinical symptoms; (ii) moderate: fever, respiratory symptoms and radiological evidence of pneumonia; (iii) severe: dyspnoea, respiratory frequency > 30/min, blood oxygen saturation ≤ 93% in ambient air and/or lung infiltrates progressing to involve > 50% of the lung within 24–48 h of admission; (iv) critical: respiratory failure, septic shock, and/or multiple organ dysfunction or failure or death. 

Specimens of clotted and heparinised blood were collected from the patients within the first 4 weeks after onset of illness. The serum and plasma were separated and stored at −80 °C until use and heat-inactivated at 56 °C for 30 min before use. 

Sera and plasma collected from Hong Kong blood donors from June to August 2017 for an influenza sero-epidemiology study were used as controls. An age-stratified panel of 200 sera and plasma were drawn from the archive, representing the age groups 16–19, 20–29, 30–39, 40–49, 50–59 and 60–69 years with 33–34 sera in each age group. A similar age-stratified panel of 472 plasma samples were also drawn. Twelve convalescent sera from a household study including endemic human coronavirus infection [6] were included as specificity controls. Seven convalescent sera from patients infected with SARS in 2003 were also included as controls. These seven sera had antibody titres of 1:20, 1:40, 1:80, 1:80, 1:160; 1:320 and 1:320 when tested by us in a micro-neutralisation test to SARS CoV.

### Cell lines

Vero E6 cells (ATCC CRL-1586) were maintained in Dulbecco's Modified Eagle Medium (DMEM) medium supplemented with 10% fetal bovine serum (FBS) and 100 U/mL of penicillin-streptomycin. Sf9 cells (*Spodoptera frugiperda* ovarian cells, female, ATCC catalogue no. CRL-1711) and High Five cells (*Trichoplusia ni* ovarian cells, female; Thermo Fischer Scientific, Waltham, United States (US), catalogue number: B85502) were maintained in HyClone (GE Health Care, Chicago, US) insect cell culture medium.

### Protein expression and purification

The RBD (residues 319–541) of the SARS-CoV-2 spike protein (GenBank accession number: QHD43416.1) were cloned into a customised pFastBac vector [7]. The RBD constructs were fused with an N-terminal gp67 signal peptide and a C-terminal His6 tag. Recombinant bacmid DNA was generated using the Bac-to-Bac system (Life Technologies, Thermo Fisher Scientific). Baculovirus was generated by transfecting purified bacmid DNA into Sf9 cells using FuGENE HD (Promega, Madison, US), and subsequently used to infect suspension cultures of High Five cells (Life Technologies) at a multiplicity of infection (moi) of 5 to 10. Infected High Five cells were incubated at 28 °C with shaking at 110 rpm for 72 h for protein expression. The supernatant was then concentrated using a Centramate cassette (10 kDa molecular weight cut-off for RBD, Pall Corporation, New York, USA). Spike RBD proteins were purified by Ni-NTA Superflow (Qiagen, Hilden, Germany), followed by size exclusion chromatography and buffer exchange to phosphate-buffered saline (PBS) [8].

### ELISA binding assay

96-well ELISA plates (Nunc MaxiSorp, Thermo Fisher Scientific) were first coated overnight with 100 ng per well of the purified recombinant RBD protein in PBS buffer. An additional plate was coated overnight with PBS buffer only and used as control to subtract non-specific serum binding to the plate, i.e. serum-specific background noise (SSBN) normalisation approach [9]. The plates coated with either purified recombinant protein or PBS were then blocked with 100 μl of Chonblock blocking/sample dilution ELISA buffer (Chondrex Inc, Redmon, US) and incubated at room temperature for 2 h. Each serum or plasma sample was tested at a dilution of 1:100 in Chonblock blocking/sample dilution ELISA buffer and added to the ELISA wells of each plate for 2 h incubation at 37 °C. After extensive washing with PBS containing 0.1% Tween 20, horseradish peroxidase (HRP)-conjugated goat anti-human IgG (1:5,000, GE Healthcare) or HRP-conjugated goat anti-human IgM (1:5,000, GE Healthcare) was added for 1 h at 37 °C. The ELISA plates were then washed five times with PBS containing 0.1% Tween 20. Subsequently, 100 μL of HRP substrate (Ncm TMB One; New Cell and Molecular Biotech Co. Ltd, Suzhou, China) was added into each well. After 15 min incubation, the reaction was stopped by adding 50 μL of 2 M H_2_SO_4_ solution and analysed on a Sunrise (Tecan, Männedorf, Switzerland) absorbance microplate reader at 450 nm wavelength. Normalised results were obtained by calculating the difference between the OD of the purified recombinant protein-coated well and the PBS-coated well.

### Microneutralisation tests

The BetaCoV/Hong Kong/VM20001061/2020 virus isolated from the nasopharynx aspirate and throat swab of a COVID-19 patient in Hong Kong was grown in Vero E6 cells. Stock virus was prepared, aliquoted and stored at −80 °C until use. The virus stock was titrated in quadruplicate in 96-well microtitre plates on Vero E6 cells in serial 0.5 log_10_ dilutions (from 0.5 log to 8 log) to obtain 50% tissue culture infectious dose (TCID_50_). The plates were observed in a phase contrast microscope for cytopathic effect (CPE) daily for 4 days. The endpoint of viral dilution leading to CPE in 50% of inoculated wells was estimated by using the Reed Muench method [10] and designated as one TCID_50_.

Serial twofold dilutions of heat-inactivated sera were made, starting with a dilution of 1:10. The serum dilutions were mixed with equal volumes of 200 TCID_50_ of SARS-CoV-2 as indicated. After 1 h of incubation at 37 °C, 35 μL of the virus–serum mixture were added in quadruplicate to Vero E6 cell monolayers in 96-well microtitre plates. After 1 h of adsorption, an additional 150 μL of culture medium was added to each well and the plates incubated for 4 days at 37 °C in 5% CO_2_ in a humidified incubator. A virus back-titration was performed with culture medium replacing serum to assess input virus dose. The CPE was read at 4 days post infection. The highest serum dilution that completely protected the cells from CPE in half of the wells was taken as the neutralising antibody titre [11]. These procedures were carried in a biosafety level 3 facility.

### Plaque reduction neutralisation tests

The PRNT was performed in duplicate using 24-well tissue culture plates (TPP Techno Plastic Products AG, Trasadingen, Switzerland) in a biosafety level 3 facility. Serial dilutions of serum samples were incubated with 30–40 plaque-forming units of virus for 1 h at 37 °C. The virus–serum mixtures were added onto Vero E6 cell monolayers and incubated 1 h at 37 °C in 5% CO_2_ incubator. Then the plates were overlaid with 1% agarose in cell culture medium and incubated for 3 days when the plates were fixed and stained. Antibody titres were defined as the highest serum dilution that resulted in > 90% (PRNT_90_) reduction in the number of plaques [11].

### Statistical analysis

We defined a sample as ELISA antibody-positive if the OD_450_ value was 3 standard deviations (SD) above the mean of the negative controls (n = 200). Correlation between serum ELISA OD, MN and PRNT_90_ antibody titres (after logarithmic transformation of antibody titres) was assessed using Pearson’s correlation coefficients. Comparison of antibody responses between disease severity groups was assessed using point biserial correlation coefficients.

### Ethical statement

The study on the COVID patients was approved by the Research Ethics Committee of the Kowloon West Cluster reference No. KW/EX-20–039 (144–27) of the Hospital Authority of Hong Kong. The study for the collection of blood donor serum and plasma was approved by the Institutional Review Board of The Hong Kong University and the Hong Kong Island West Cluster of Hospitals (IRB reference number UW16–254).

## Results

Fifty-one sera from 24 patients were included in the investigation, 17 of these patients had two to four sequential serum samples available for study. Five of the 24 patients (28–63 years-old) had mild disease, 12 (25–80 years-old) were moderate, three (60–72 years-old) were severe and four (56–64 years-old) were critically ill.

None of 200 control sera collected before the emergence of COVID-19 had any MN or PRNT_90_ antibodies to SARS-CoV-2 at a screening dilution of 1:10. None of the convalescent sera from patients in the household study with RT-PCR-documented coronavirus 229E (n = 2), NL63 (n = 3), OC43 (n = 4) or HKU-1 (n = 3) infections were positive in the MN assay. There were insufficient volumes of these to test in the other assays. In order to optimise sensitivity of the ELISA assays as a screening test, we set the cut-off for a positive result as the mean +3 SD of negative control sera (n = 200). The corresponding cut-off was OD >0.40 for the IgG ELISA and OD >0.67 for the IgM ELISA. None of 200 control blood donor sera were above the cut-off for either the IgG ELISA or IgM assays. None of the seven SARS convalescent sera were positive in the ELISA IgG, MN or PRNT_90_ assays, but three were weakly positive in the IgM assay.

**Figure 1:**
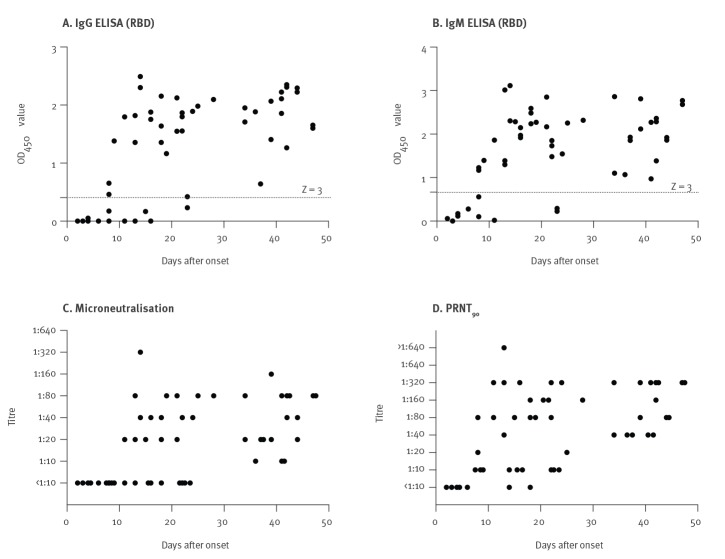
Antibody responses of the COVID-19 patient cohort in relation to duration of illness, Hong Kong, March 2020 (n = 24 patients, 51 sera)

COVID-19: coronavirus disease; OD: optical density; PRNT: plaque reduction neutralisation test; RBD: receptor-binding site.

Figure 1 shows the results from the whole patient cohort in relation to duration of illness on the ELISA IgM and IgG, MN and PRNT_90_ assays. None of four sera collected within the first 4 days of illness were positive in any of the assays. None of these sera had detectable viral RNA in the serum specimen. Of the six sera collected 5–9 days after onset, three were positive for IgM and IgG in ELISA, none were positive by MN and four, including all three sera positive in ELISA, were positive by PRNT_90_. Of 14 sera collected 11–18 days after onset, 13 were positive for IgM, 10 for IgG, nine in MN and 13 in PRNT_90_, and one serum was negative in all four assays. Of 11 sera collected from 19–28 days after onset, nine were positive for both IgM and IgG, seven in MN and all 11 in PRNT_90_. The 12 patients who were sampled between Day 29 and 42 were all positive in all four assays. The MN antibody titres in those who had become seropositive ranged from 1:10 to 1:320, and PRNT_90_ titres ranged from 1:10 to 1:1,280 (the highest serum dilution tested). We found that the PRNT_90_ titres in serum were approximately fourfold higher than those in the MN assay when the conventional 100 TCID_50_ virus challenge was used in the MN test.

In the ELISA assays, the mean OD_450_ value in the controls +3 standard deviations above the mean (Z score > 3) was considered as positive.

By grouping mild/moderate cases and severe/critical cases, we assessed the correlation of antibody responses with disease severity in the sera collected after Day 14. The log of neutralisation titres (PRNT_90_) (point biserial correlation coefficient = −0.05; p value = 0.79) and ELISA IgM OD (point biserial correlation coefficient = 0.08; p value = 0.69) were not correlated with disease severity but severe/critical cases had higher serum ELISA IgG OD than the mild/moderate cases (point biserial correlation coefficient = 0.37; p-value = 0.049).

**Figure 2:**
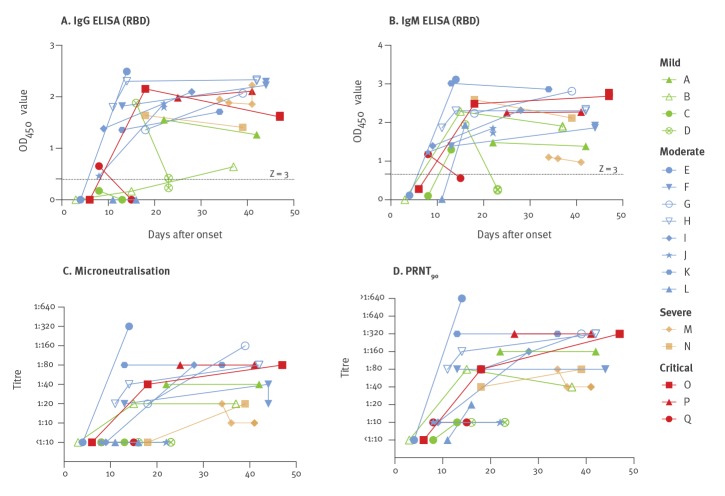
Kinetics of antibody response in individual patients with SARS-CoV-2 infection by days after illness onset, Hong Kong, March 2020 (n = 17 patients)

PRNT: plaque reduction neutralisation test; SARS-CoV-2: severe acute respiratory syndrome coronavirus 2.

**Figure 3:**
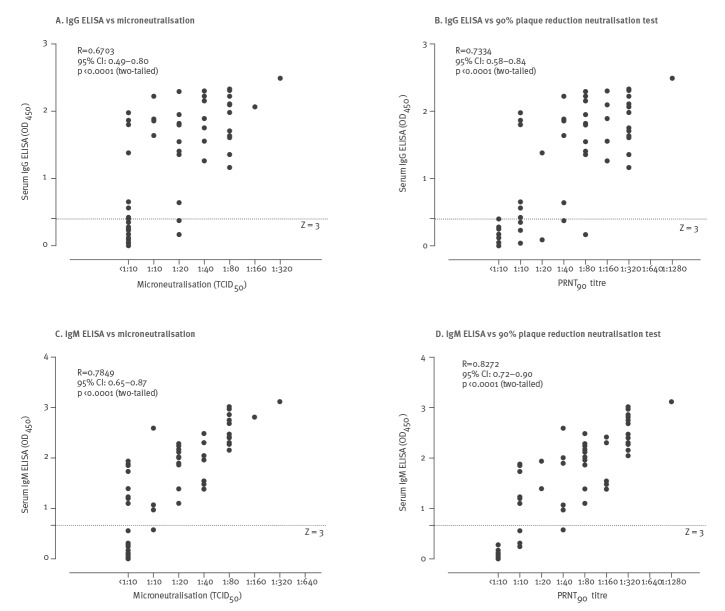
Correlation between the ELISA test results and microneutralisation and plaque reduction neutralisation results in COVID-19 patients, Hong Kong, March 2020 (n = 24 patients, 51 sera)

CI: confidence interval; COVID-19: coronavirus disease; OD: optical density; PRNT: plaque reduction neutralisation test; R: Pearson coefficient; TCID: tissue culture infectious dose.

Figure 2 shows the antibody responses for these four assays in the 17 individual patients from whom we had sequential serum samples. Most of the patients, including those with mild non-pneumonic illness, developed detectable MN and PRNT_90_ antibody responses, provided they had sera collected beyond 28 days after illness. Figure 3 shows the correlation between the MN or PRNT_90_ titres and IgG or IgM ELISA OD.

In the ELISA assays the mean OD_450_ value in the controls +3 standard deviations above the mean (Z score > 3) is considered as positive.

Pearson correlation analysis with p value ≤ 0.05 was considered as significant.

**Figure 4:**
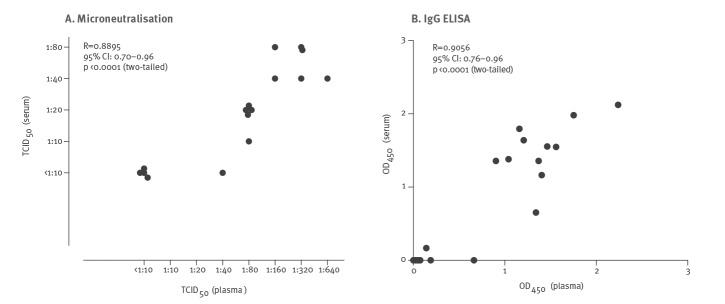
Correlation between microneutralisation and IgG ELISA in pairs of plasma and serum specimens collected on the same day from COVID-19 patients, Hong Kong, March 2020 (n = 16 pairs of serum and plasma)

CI: confidence interval; COVID-19: coronavirus disease; OD: optical density; R: Pearson coefficient; TCID: tissue culture infectious dose.

Sixteen of the tested sera had parallel heparinised plasma samples collected on the same day. Figure 4 shows the correlation of results in IgG ELISA and MN assays in plasma and serum collected on same day from the same patient. Plasma samples consistently had 4- to 16-fold higher MN titres than the corresponding serum while none of 472 control plasma samples had any detectable MN antibodies at a screening dilution of 1:10. 

Pearson correlation analysis with p value ≤ 0.05 was considered as significant.

We hypothesised that heparin or other components within heparin-anticoagulated blood collection tubes may block virus infectivity, thus reducing the infectivity of the TCID_50_ challenge dose of the virus in the MN assay and increasing the sensitivity of the MN assay for antibody. We therefore added tissue culture medium (DMEM with 2% FBS) into heparin blood collection tubes to mimic the volume of blood to be collected in each tube (heparin medium). After mixing, the medium was withdrawn and used as diluent in titrating stock SARS-CoV-2 virus of known titre in half-log_10_ dilution steps. Culture medium without exposure to the heparin blood collected tubes was used for control dilution series. We observed a 1–1.5 log_10_ reduction in TCID_50_ when the virus was diluted in the heparin medium compared with the control medium. We also carried out titrations of three sera with known MN antibody titres of 1:40, 1:80 and 1:80, with the serum dilutions carried out in parallel in heparin medium or control medium. The antibody titres in the sera diluted in the heparin medium were 1:160, 1:320 and 1:320 respectively. These results suggested that the higher antibody titres obtained with plasma were due to a lower effective virus challenge dose.

Further, we examined the effect of reducing the viral challenge dose on serum MN titres. We titrated three sera, collected from three different patients on Day 4, 21 and 22 after onset of illness, using challenge doses of 100, 50 and 25 TCID_50_ in the MN assay. These three sera had previously had MN antibody titres of < 1:10, 1:40 and 1:80 when titrated using the conventional MN virus challenge dose of 100 TCID_50_ but titres of < 1:10, 1:160 and 1:320 in the PRNT_90_ assay. The antibody titres of the serum at Day 4 after illness onset remained negative irrespective of the virus challenge dose down to 25 TCID_50_. The titres of the other two sera progressively increased twofold with each reduction of the TCID_50_ challenge dose of the virus and became comparable with the PRNT_90_ titre. None of the control sera gave had any false positive ‘neutralisation’ when tested with a TCID_50_ of 50 or 25.

## Discussion

Our primary objective was to evaluate and validate a screening and confirmation strategy for large scale sero-epidemiolgical assays to assess infection attack rates in the population. Our strategy was to use an IgG ELISA targeting the SARS-CoV-2 spike RBD as a screening assay, confirming any positive results by PRNT_90_ assays. Even though the RBD ELISA assay was specific in our evaluation reported here, a confirmatory test with neutralisation would be good practice for the studies envisaged. Even more importantly, not all RBD ELISA-positive samples may have neutralising activity. While a positive RBD ELISA result, even if specific, provides evidence of prior infection with SARS-CoV-2, it is no assurance of protective immunity, whereas the presence of neutralising antibodies would provide greater assurance of protection. However, more research is needed on the correlates of protection in all these serological assays. Our results on the comparative sensitivity of the MN and PRNT_90_ test suggest that PRNT_90_ is the confirmatory assay of choice, at least until a fully validated pseudotype neutralisation assay is available. The RBD ELISA can be completed in a day while the PRNT_90_ neutralisation confirmation takes 4 days more and two tests require ca 100 μl of serum. 

The general population has detectable antibodies to a range of endemic human coronaviruses such as 229E, OC43, HKU1 and NL63, and this seroprevalence increases with age [12,13]. In order to optimise the sensitivity of the SARS-CoV-2 RBD IgG ELISA as a screening assay, we set the cut-off for a positive result as 3 SD above the mean of negative control sera. None of the control sera had IgM or IgG ELISA OD values above the cut-off. Similarly, none of the negative control sera gave any reactivity in the MN or PRNT_90_ assays. Convalescent sera from patients with RT-PCR-confirmed 229E, OC43, NL63 and HKU1 infections were negative in MN assays to SARS-CoV-2, further confirming the specificity of the MN assay, but there was insufficient serum available to test these sera in PRNT_90_ or ELISA assays. The SARS-CoV-2 ELISA IgG, MN and PRNT_90_ assays showed no detectable cross-reactivity even to the closely related SARS-CoV in convalescent sera from SARS patients. We therefore conclude that the ELISA IgG, MN and PRNT_90_ antibody assays are specific for SARS-CoV-2. Extensive experience with MN and PRNT_90_ assays for MERS-CoV [11] provides further reassurance that these neutralisation assays are specific, discriminate between different human coronaviruses and can be used for confirmation of sera positive in the screening ELISA.

When these assays were evaluated in a cohort of RT-PCR confirmed SARS-CoV-2-infected patients, we found that the patients progressively developed detectable antibody after 5 days of illness in the ELISA IgM and IgG and PRNT_90_ assays and after 10 days of illness in the MN assay. All sera collected 29 or more days after onset of illness were antibody-positive in all four serological tests, findings similar to another recent report [14]. The OD levels in the IgG ELISA were statistically significantly higher in severe/critical cases, a finding also reported by others [14,15] but as the investigated number of patients remains small, these findings need to be confirmed in a larger patient cohort.

The type of blood specimens that may be available for large-scale sero-epidemiological studies may vary with the study design. While serum is the conventional specimen used in such studies, plasma may be available from residual blood submitted for other laboratory tests such as those sent for clinical biochemistry or haematology investigations. We therefore evaluated in the ELISA IgG and MN assay a small number of heparin plasma samples available from these patients, collected in parallel with the serum samples. There was good correlation in the IgG ELISA results between serum and plasma. However, it was notable that the MN titre in plasma was 4–16-fold higher that than detected in the concurrent serum. We showed that this was due to the heparin or another component in the heparin blood collection tube that contributed to reducing the effective TCID_50_ of the virus challenge dose in MN assays. Thus, caution is needed in the interpretation of neutralisation results from heparin plasma. None of the negative control plasma samples tested gave any detectable neutralisation of the virus at a screening dilution of 1:10. Nevertheless, if neutralisation is going to be used for confirmation, it would be important that heparinised plasma is avoided because of unpredictable effects on the effective virus inoculum. We have not investigated the effect of ethylene-diamine-tetra-acetic acid (EDTA)-anticoagulated plasma.

We found that the PRNT_90_ titres in serum were higher than those in the MN assay when the conventional 100 TCID_50_ virus challenge was used in the MN test. Reduction of the virus challenge dose to 50 or 25 TCID_50_ in serum-MN assays substantially increased sensitivity but may compromise specificity.

We have a very stringent cut-off (3× standard deviation of the mean) in our ELISA assay to minimise detection of cross-reactive antibody in the population and reduce false positive results. Since screen-positive sera are being subjected to a confirmatory test, the use of a 2× standard deviation cut-off may increase sensitivity of the screening ELISA test at the expense of specificity, to allow more sera to be picked up for confirmation by PRNT_90_ testing. These trade-offs may need to be optimised as a screening programme progresses. 

Although our study was not designed primarily to assess serology as a diagnostic test, our results indicate that none of the four serological tests evaluated permitted detection of SARS-CoV-2-specific antibodies early in the course of infection and was only reliably positive longer than 4 weeks after onset of illness. Thus the use of serology for diagnosis of acute disease must be viewed with caution and RT-PCR for virus detection remains the method of choice.

Limitations of our study include the small number of patients and sera investigated and the lack (as yet) of longer time of follow-up of these patients to determine the duration of these antibody response. Furthermore, while we have demonstrated serological responses in patients with mild non-pneumonic COVID-19 illness, we also require more data from asymptomatic infections to define how commonly such infections lead to an antibody response. Lack of a panel of convalescent sera from patients with RT-PCR-confirmed human coronaviruses 229E, OC43, HKU1 and NL63 is also a limitation.

## Conclusion

The screening with SARS-CoV-2 IgG ELISA followed by PRNT_90_ confirmation is a reliable approach for large-scale sero-epidemiological studies which are crucial to assess infection attack rates in the population and to accurately define disease severity [5] and herd immunity. We have previously shown that large-scale sero-epidemiological studies can provide population infection attack rates in near real time [16].
